# Creating Locally Relevant Health Information

**DOI:** 10.1371/journal.pmed.0020046

**Published:** 2005-03-29

**Authors:** Isabel Carter

## Abstract

Lack of health information marginalises the poor and prevents them from making informed decisions. What we need, argues Carter, is to promote health in ways that are accepted by local communities

## Background

It is an ongoing challenge to share health information with resource-poor communities that is locally relevant and owned by the communities themselves. Too often, new information brought to these settings is seen as coming from “outside” and therefore as having little local relevance. People may look with suspicion at those who bring such information. Many factors—the background, attitudes, clothing, employers, and the language of those who bring information—may have more impact on the way new ideas are received than the actual relevance of the ideas themselves. We have only to consider our own attitudes to politicians for an example of how such factors influence our receptivity to information.

When health information from outside the community goes against deeply held beliefs and attitudes about personal and sexual matters, this challenge becomes still greater. Providing opportunities for people to discuss the impact of HIV and AIDS on their communities in a relaxed and open manner is key to enabling people to engage in potentially lifesaving discussion and attitude change.

But as positive a step as open discussion is, unless poor people can access and accept the information they need, they will not be able to make informed decisions regarding their lives and future. Ignorance about the impact of HIV and AIDS and how the virus is transmitted is potentially life-threatening; we urgently need to raise awareness in ways that are accepted by local communities.

## PILLARS: Partnership in Local Language Resources

**Figure 1:**
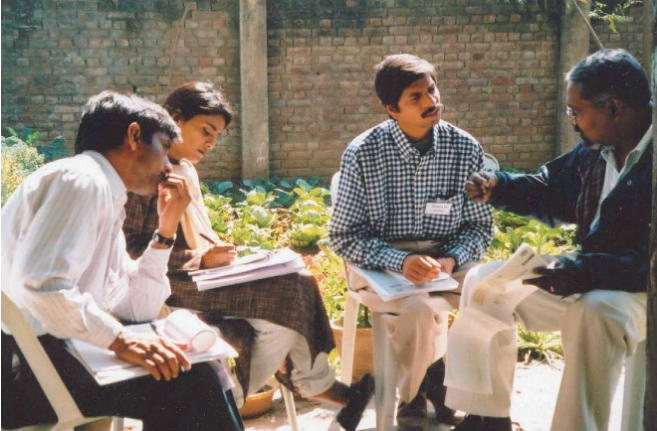
Participants Discussing a Topic on Leadership Styles during a PILLARS Workshop in Delhi

Between 1995 and 1999, Tearfund UK, a Christian community development and disaster relief charity, conducted research into the flow of information at grassroots level in Uganda and Ghana, with the support of the education division of the United Kingdom Department for International Development [1]. The findings highlighted the importance of small groups in sharing information, the lack of relevant printed materials for the poor, and the need for end users to be involved in the creation of relevant printed information in their own languages. These findings have now been translated into practical action through Partnership in Local Language Resources (PILLARS). PILLARS guides provide small community groups with simple printed information, written in local languages, on community development issues such as nutrition, food security, micro-credit, HIV and AIDS, and community mobilisation (see www.tilz.info/resources). These guides are not seen by communities as information from an outside entity, but rather as locally consolidated information on relevant issues for groups to discuss in their regular meetings. Rather than acting as passive recipients of information, group members can bring useful experience, knowledge, and insights into the discussion (Figure 1).

Each guide contains 20–24 topics, with illustrations, text, and discussion questions. Any group that meets on a regular basis can set aside time to read through and discuss one of the topics. A trained leader is not required, though it helps if someone in the group knows how to facilitate discussion. The guides build on existing knowledge and experience shared among group members.

## Empowering the Community

The ultimate goal of PILLARS is to empower community groups in developing nations by building their capacity for collective learning, consensus-building, and subsequent action. Use of the guides restores their right to receive and share information in their own tongue and to participate in the development of their communities. The generation, use, and distribution of information in local languages encourages and gives confidence and value to marginalised groups. The discussion process helps groups manage their own change and engage in local decision-making processes.

Guides are now available on nine subjects relating to community development. They are designed for ease of translation into any language using a CD-ROM with design and text files. Pages or illustrations can be contextualised to meet local needs, and participatory bible studies are included at the back of the guides for faith-based groups to use.

A further aspect of PILLARS is that development workers can be equipped to translate and write new guides over the course of three workshops. During the first two workshops, participants learn skills in translation, reviewing, and field testing. In the final workshop, participants write their own guide and plan for future sustainability. Training in facilitation skills and participatory techniques equips participants to use the guides.

This process has been piloted in southern Sudan, Ethiopia, Nigeria, Burkina Faso, Brazil, and Myanmar. In Myanmar, development workers produced guides in Burmese and then replicated the training with a further 13 language groups, generating considerable energy and empowerment. A facilitator commented: “We have so many languages in our country. Through this programme, people are encouraged to value their culture and to share useful information about development with the community.” In Ethiopia, participants wrote a guide on “harmful traditional practices”. Training has also been conducted there with a refugee community from southern Sudan, helping them plan for repatriation.

A recent evaluation, led by Dr Clinton Robinson of PILLARS in Myanmar, Brazil, and Ethiopia, revealed a dearth of written information in the languages of minority groups. Access to information and to the media was generally low. The evaluation found that improving access to simple, relevant and practical information in local languages increased people's self-confidence and their ability to make positive change. It commented on the benefits of the emphasis PILLARS places on collective learning rather than on individual reading.

## Discussion-Based Learning on HIV and AIDS

**Figure 2:**
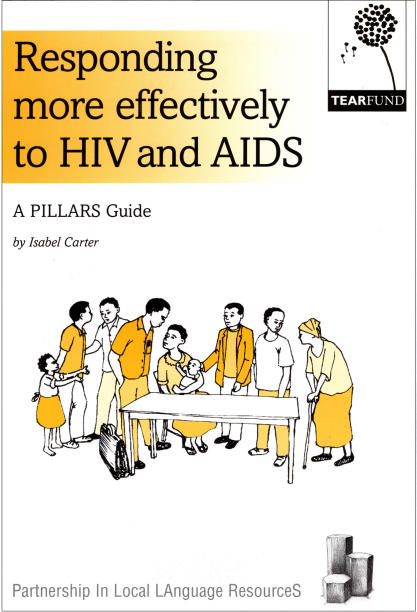
A Recent PILLARS Guide

A new guide, *Responding More Effectively to HIV and AIDS*, is now available (Figure 2) [2]. With funding from Development Corporation Ireland, this guide is being translated into a further nine languages: French, Spanish, Portuguese, Hindi, KiSwahili, Amharic, Khmer, Kinyarwandan, and Chinese.

The guide first gently challenges misconceptions to ensure that people have the correct facts about HIV/AIDS and how the virus is passed on. Issues raised include traditional practices that might spread HIV, the need for HIV testing and accompanying counselling, and the needs of children who lose their parents from HIV/AIDS. The guide encourages discussion of how to talk about sexual issues with children, with partners, and within faith-based teaching. The burden that can fall upon carers who respond to the needs of families living with HIV/AIDS can be immense, and several topics encourage people to discuss this. The guide also addresses relevant and challenging questions, including who provides the caring, who else could help, what support systems are available, and how the local community can increase its support. Recent advances in antiretroviral therapy and the latest advice regarding breastfeeding by mothers with HIV are also covered.

At a recent workshop in Nairobi, staff from Sudan gained facilitation skills and learned techniques to share information effectively. They worked in small groups to develop simple role-plays to introduce topics from the HIV and AIDS guides. Though few of the participants had used this method before, they produced some amazingly powerful role-plays that provided a very effective introduction to the group discussion and learning that followed.

## The Local Production of Information

A number of organisations have developed tools and training to help with the local production of information (Box 1). PILLARS differs from these approaches, both in its focus on providing information for discussion-based learning targeted at grassroots community groups, and in providing technical support and design files to simplify the translation process.

**Box 1. Tools for the Local Production of Information**

- Agricultural kits produced by the International Institute for Rural Reconstruction (www.iirr.org).
- Shell booklets produced by the Summer Institute of Linguistics (www.sil.org), often used as literacy primers in local languages.
- The REFLECT approach (www.reflect-action.org), a method of increasing literacy using participatory techniques. Each literacy circle produces its own learning materials, analysing its own local community and its immediate circumstances.
- The STAR (Stepping Stones and Reflect) Initiative (www.healthcomms.org/pdf/STARsummary.pdf), which provides draft guidelines that support communities or organisations to analyse and tackle issues that affect them, from agriculture to war, in the context of HIV and AIDS.
- The Quest manual, from Healthlink Worldwide (www.healthlink.org.uk/consult/quest.html), a tool to support organisations to develop their capacity to produce effective communication and information resources.

PILLARS guides have now been translated into more than 30 different languages and are being used with basic literacy programmes and training workshops, and as discussion-based materials for women's, farmers', and credit groups. They offer a simple, yet potentially very effective method of sharing health messages. The use of print means that such messages can be used over the long term and widely distributed.

A free copy of the HIV and AIDS guide for groups in resource-poor nations is available from E-mail: roots@tearfund.org.

## Abbreviation

PILLARS: Partnership in Local Language Resources
